# Charge of clustered microparticles measured in spatial plasma afterglows follows the smallest enclosing sphere model

**DOI:** 10.1038/s41467-021-23604-z

**Published:** 2021-08-03

**Authors:** B. van Minderhout, J. C. A. van Huijstee, R. M. H. Rompelberg, A. Post, A. T. A. Peijnenburg, P. Blom, J. Beckers

**Affiliations:** 1 Department of Applied Physics, Eindhoven University of Technology, Eindhoven, The Netherlands; 2 VDL Enabling Technologies Group, Eindhoven, The Netherlands

**Keywords:** Applied physics, Plasma physics

## Abstract

The plasma-induced charge of non-spherical microparticles is a crucial parameter in complex plasma physics, aerosol science and astrophysics. Yet, the literature describes this charge by two competing models, neither of which has been experimentally verified or refuted. Here we offer experimental proof that the charge on a two-particle cluster (doublet) in the spatial afterglow of a low-pressure plasma equals the charge that would be obtained by the smallest enclosing sphere and that it should therefore not be based on its geometrical capacitance but rather on the capacitance of its smallest enclosing sphere. To support this conclusion, the size, mass and charge of single particles (singlets) and doublets are measured with high precision. The measured ratio between the plasma-afterglow-induced charges on doublets and singlets is compared to both models and shows perfect agreement with the predicted ratio using the capacitance of the smallest enclosing sphere, while being significantly dissimilar to the predicted ratio based on the particle’s geometrical capacitance.

## Abstract (web summary)

Here the authors report measurements of the charge ratio and mass of two-particle clusters and single microparticles in the spatial plasma afterglow. The insights contribute to the general understanding of non-spherical particle charging in ionized gasses.

## Introduction

The charge and charging of nonspherical or clustered nano-to-micrometer-sized particles in partly ionized media is a key parameter in research fields such as complex plasma physics, astrophysics, and aerosol science. In this paper, the term clustered particles refers to aggregates of individual particles. In complex plasma physics, studying the fundamental interaction between particles and plasma, the nonspherical particle charge drives the coagulation and aggregation of particles that are synthesized within the plasma discharge^1,2^. Therefore, cylindrically shaped particles^3–9^, spheroid-shaped particles,^10,11^ and aggregates^12^ have attracted special interest. In astrophysics, the charging of clustered particles^13^ is studied especially^14–19^, since these are commonly found in protoplanetary disks where planet formation takes place^17–20^. The charge of clustered particles seems heavily dependent on their morphology, which in turn impacts the particles’ accretion process^17^. In aerosol science, extensive experimental and computational research is reported on the charging of nonspherical particles at atmospheric pressures^21–26^, since particle charge is of vital importance in determining particle size in differential mobility analyzers.

From an application point of view, understanding the collection of charges by nonspherical particles from their environment has major implications. Plasma-assisted contamination control strategies^27^ may have high potential to tackle the challenge of particle contamination, both with regard to ultraclean systems in the semiconductor industry^28^ as well as to lowering worldwide air pollution produced by combustion engines^29,30^. The principle of these plasma-assisted control techniques stems from the ability of plasma to charge the nonspherical particles and to affect their trajectories by either plasma-induced^31^ or externally applied electric fields. Since the latter could be shielded in the plasma bulk, particle charging in the plasma afterglow (with pure diffusion charging in the limiting case) is of the utmost importance for the two respective applications.

In medical applications, the charge and morphology of nanoparticles (NPs) used in medications play a vital role in drug delivery^32–34^. Positively charged NPs improve the efficacy of imaging, gene transfer, and drug delivery, while they also have higher cytotoxicity^35^. The latter shows that charge control of these NPs is crucial and can be achieved in the spatial plasma afterglow, as has been recently demonstrated^36,37^.

It thus seems clear that understanding the charging mechanism of nonspherical particles embedded in media where free charges are present is of high importance in relevant fundamental and applied fields. Apart from this importance and its potential impact, one more thing stands out in these examples: the literature provides competing theories for calculating and predicting the charge on nonspherical and clustered particles.

On the one hand, the plasma-induced charge is described by equivalent sphere models determining the equivalent particle capacitance, of which two variants exist. Matthews et al. reported that, as long as the plasma currents to the particle surface dominate, the charge of clustered particles could be described by the charge that the sphere with the orientation-averaged equivalent radius *R*_*σ*_ would obtain^17,38^. The morphology of the clustered particle determines the value of *R*_*σ*_, distinguishing between filigree and compact-shaped particles. Asnaz et al. showed that, in the particular case of the anisotropic plasma sheath, the capacitance of the smallest enclosing sphere correctly predicts the charge for tetrapods^39^.

In complex plasma physics, however, the charge on nonspherical particles such as cylinders^3–9^ and spheroids^10,11^ is commonly calculated or simulated based on the particle’s geometrical capacitance^40^, opposed to the equivalent particle capacitance used in the equivalent sphere models. The simulations of Sukhinin et al. indicated that the particle charge is described by the geometrical capacitance if the charged plasma species are in the collisionless and weakly collisional regime^40^ and that this might not be the case for the collisional regime as under investigation in this paper.

The description of particle charge using the geometrical capacitance has provided important insights in the charging of spherical particles in temporal plasma afterglows, where the Debye length can exceed the plasma bulk value^41–43^. In aerosol science, a uniform theory describing the nonspherical particle charge has equally been lacking^24–26^ until Li and Gopalakrishnan recently proposed a unifying experimentally validated theory^44^.

Thus far, it has remained unclear which theoretical framework should be used for low-density plasmas, such as the plasma afterglow under investigation here, since experimental data verifying or refuting either of the models have been nonexistent.

Here, we report accurate charge measurements of clustered microparticles in a plasma afterglow, thereby proving that the charge on two-particle clusters (doublets) is correctly described by the charge on the smallest enclosing sphere. The structure of this paper is as follows: first, the particle charge distributions of single microparticles (singlets) and doublets are presented and discussed in the “Results” section, and the measured ratios between the respective distributions are compared with the ratios predicted by the various models in the “Discussion” section. Finally, an overview of the experimental setup is given, followed by the theoretical framework of the applied in situ cluster detection method in the “Methods” section, where the drag and torque appear to be essential.

## Results

The results of this paper are divided into two sections: the in-situ determined cluster sizes and the presentation and comparison of the measured charge distributions of singlets and doublets.

### Cluster sizes

The technique used to differentiate between single and clustered microparticles is based on the difference in neutral drag that the particles experience, which results in distinct settling velocities. This section presents measurements and analysis of the vertical settling velocity *v*_y_, from which the particle mass and cluster size are ascertained.

The variation in individual particle mass was obtained from the measured settling velocity *v*_y_ using the neutral drag described in section 4 (Eq. (3)). Since gravity and drag are the dominant vertical forces, the radius of each singlet was obtained from

1 $${r}_{{{{\rm{p}}}}}=\frac{{v}_{{{{\rm{y}}}}}\delta {\rho }_{{{{\rm{n}}}}}{v}_{{{{\rm{th}}}},{{{\rm{n}}}}}}{{\rho }_{{{{\rm{p}}}}}g},$$

where *ρ*_n_ represents the mass density of the argon gas, *v*_th,n_ the thermal velocity of the gas particles, *ρ*_p_ the particle’s average mass density, and *g* the gravitational constant. Moreover, the coefficient *δ* accounts for the microscopic mechanism of the atom-particle collisions (see for more details the cluster detection description in the “Methods” section). The peak of the measured vertical velocity distribution for singlets stood at 4.7 × 10^−2^ m s^−1^, as will be shown at the end of this section. In section 4, the morphology of the used particles’ outer layer is discussed using SEM analysis. Based on the observed surface roughness and the assumption that the particle is thermally nonconductive (the conductive coating is small compared to the insulating core), *δ* = 1.442 must hold^45^, which states that the microscopic mechanism of the argon collisions at the particles’ surface (see Eq. (3)) is described by diffuse collisions.

**Fig. 1:**
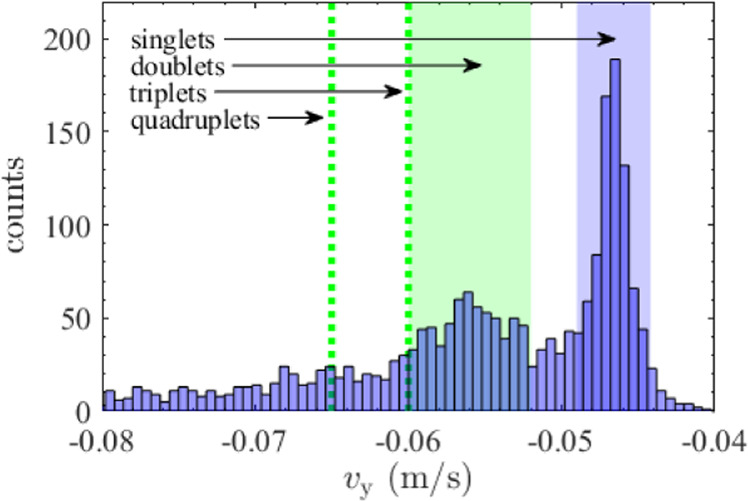
Vertical velocity distribution. Histogram of the measured vertical velocity of all detected particles. The green band indicates the reported literature values of doublet velocities (see Table 1), which perfectly match the doublet peak observed in the velocity distribution. The green dotted lines show the predicted average vertical velocities of the triangular triplets and tetrahedral quadruplets. The blue band indicates the selected velocity range of the singlets.

Using the mean supplier-provided radius of 2.5 μm and assuming the argon gas temperature to equal 293 K, the rightmost peak in Fig. 1 was fitted using Eq. (1) rewritten for the singlet vertical settling velocity *v*_s_, resulting in *ρ*_p_ = 1.61 × 10^3^ kg m^−3^. The variations in particle size were corrected for each individual particle based on the measured vertical particle velocity (as explained in the previous paragraph). The fitted particle density stood above the density of pure melamine formaldehyde (MF) (*ρ*_MF_ = 1.51 × 10^3^ kg m^−3^), and was lower than the average particle density expected for particles coated with a pure silver layer of ~100 nm. The latter was considered reasonable based on the observed porosity in the silver coating as described in section 4. Using Eq. (1), the individual particle mass could easily be obtained using ${m}_{{{{\rm{p}}}}}=4/3\pi {r}_{{{{\rm{p}}}}}^{3}{\rho }_{{{{\rm{p}}}}}$, assuming the average particle density *ρ*_p_ to be constant.

In order to evaluate the cluster orientation with respect to the flow, the torque *τ* acting on a cylinder was estimated (Eq. (6)). From classical mechanics, it follows that the solution for *β* = 0 is stable, whereas the one for *β* = *π*/2 is unstable, i.e., the clusters align their long axis with the flow. Moreover, using the moment of inertia of a cylinder *I*_c_, it could be estimated that this alignment takes place in *~t*_c_ = *I*_c_/*τ* ≈ 10 ms.

Knowing the cluster orientation, the ratio of cluster (*v*_c_) to singlet (*v*_s_) velocity could be obtained by equating the drag force calculated using Eqs. (3) and (4) to the gravitational force on the particles. Hence

2 $$\frac{{v}_{{{{\rm{c}}}}}}{{v}_{{{{\rm{s}}}}}}=\frac{{N}_{{{{\rm{p}}}}}^{1/3}}{{\chi }_{\nu ,\parallel }},$$

where the subscript ∥ indicates an orientation parallel to the flow and *N*_p_ is the number of particles the cluster consists of. In the field of aerosol science, several studies^46–50^ reported particle parameters *χ*_*c*_ and *ϕ* for parallel-oriented clustered microparticles, which are shown in Table 1 together with the corresponding *v*_y_ for each cluster size (calculated using Eqs. (5) and (2)).

**Table 1:** Particle parameters and the resulting vertical velocity.

	*χ*_*c*,∥_	*ϕ*_∥_	${v}_{{{{\rm{y}}}}}\left(1{0}^{-2}{{{\rm{m}}}}\ {{{{\rm{s}}}}}^{-1}\right)$
Doublet	(1.02−1.025)	(1.21−1.384)	(5.2−6.0)
Triplets	(1.08−1.094)	(1.41−1.491)	(5.9−6.2)
Quadruplets	1.12	1.59	6.5

Particle parameters and the resulting *v*_*y*_ for doublets, triangular triplets, and tetrahedral quadruplets oriented parallel to the flow^46–50^.

Figure 1 shows the measured vertical velocity *v*_y_ of all detected particles (singlets and clusters). The distribution shows two distinct peaks. The first, centered around 4.7 × 10^−2^ m s^−1^, corresponds to the singlets, and the second, centered around 5.6 × 10^−2^ m s^−1^ to the doublets. The measured doublet peak corresponds to the calculated range (see Table 1), which is indicated by the green band. The light blue band indicates the selected velocity range for the singlets. The expected peaks of the triplets and quadruplets are indicated by green dotted lines. Evidently, no distinct peaks are visible at these velocities. Thus, too few triplets and quadruplets were detected for statistical analysis, and these cluster sizes were therefore not considered in the analysis.

**Fig. 2:**
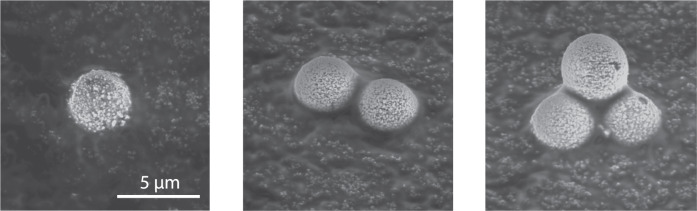
SEM images of (clustered) microparticles. Three SEM images of the observed (clustered) microparticles including a scale bar which applies to all three pictures. The bottom particles of the triplet (outer most right image) appear smaller than the top particle because these were slightly pressed into the adhesive carbon tape caused by the collection method.

Figure 2 presents three SEM images of the injected (clustered) microparticles, which were collected after passing the plasma discharge. These images show the presence of singlets, doublets, and triplets among the particles injected. A triangular configuration of the triplets was expected, since this configuration proved more stable than a string alignment^13^.

To determine the doublet mass from its measured *v*_y_, the average *χ*_*ν*,∥_ from Table 1 was used. The following section takes into account the variation in reported literature values for the uncertainties in the measured particle charge distributions.

### Charge distributions

**Fig. 3:**
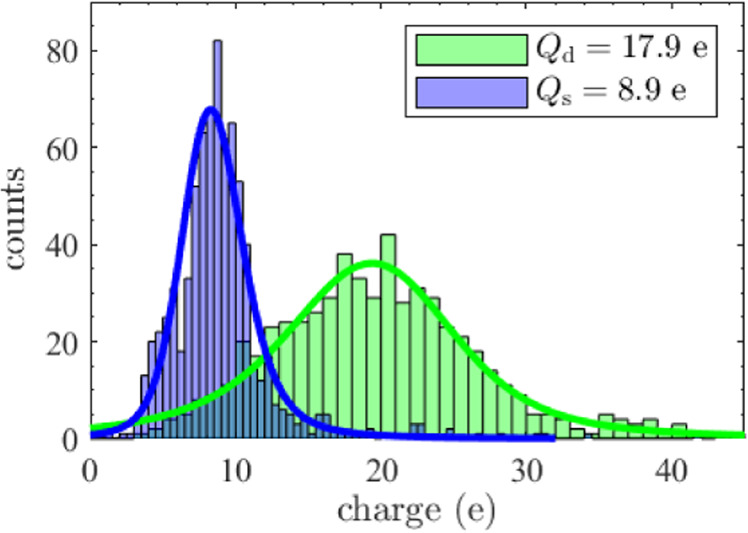
Charge distribution single particles and two-particle clusters. Histograms of the measured charge distribution of singlets (blue) and doublets (green) together with their mean positive charge. The singlet and doublet distributions consist of 761 and 611 particles respectively, of which 4 singlets and 42 doublets have a charge outside the axis limits, within the range (−66 − 84)e. Although not plotted here, these data points are included in further analysis. The average singlet charge *Q*_s_ and the average doublet charge *Q*_d_ are indicated in the legend.

Using the differentiation between singlets and doublets based on the particle settling velocity, as explained in the previous section, the charge distribution of both singlets and doublets could be measured independently. In addition to cluster size detection, variations in particle mass were taken into account based on the vertical settling velocity. Figure 3 shows the charge distribution of singlets and doublets, together with a fitted Stable distribution (also known as the Lévy alpha-stable distribution, of which the Gaussian distribution is a special case) and the corresponding mean charge. The mean charge was *Q*_s_ = 8.9 e for singlets and *Q*_d_ = 17.9 e for doublets. Thus, the ratio of the doublet to singlet charge was *R* = 2.0 ± 0.2. The uncertainty in this ratio was determined from the confidence interval of the parameters of the fitted distributions, as will be discussed in the next section.

## Discussion

The measured singlet and doublet charges were both positive, whereas the particle charge in the plasma bulk is known to be highly negative^51,52^. With regard to the late spatial afterglow, Couëdel et al. have shown that the particle charge can indeed become positive and is determined by the transition from ambipolar to free diffusion^41^. It is likely that in our experiments, the externally applied electric field influenced the latter transition. Positively charged particles in afterglows were also reported in several other studies^42,53–57^. Since the photon energy of the laser system was below the work function of silver^58^, photodetachment was assumed to be negligible. Furthermore, due to the low gas (and particle) temperature combined with the low electric field strength, thermionic emission^59^, field emission,^60^ and secondary electron emission^61^ were expected to have had a negligible influence on the particle charge.

The uncertainty in the mean singlet charge was obtained by taking half of the 95% confidence interval of the fitted Stable distribution. Overall, the mean of the distribution obtained for the singlet charge stood at  (8.8 ± 0.4) e. The uncertainty in the doublet charge was predominantly determined by the spread in particle parameters reported in the literature (see Table 1) and therefore the found mean of the distribution for the doublet charge was (18.5 ± 1.7) e. A possible error in particle mass, which could only have led to variations in the reported absolute charge values rather than in the reported charge ratio, was expected to be significantly smaller than the spread in particle parameters. Summarizing, the ratio of the means of the fitted charge distributions through the doublet and singlet data sets was *R* = 2.1 ± 0.2. Adopting the same uncertainty for the ratio of the mean doublet to mean singlet charge gave *R* = 2.0 ± 0.2.

As stated in the “Introduction” section, the charge on clustered particles has been described by two different models in the literature. No experimental proof has yet been provided regarding which of the two models would be correct for spatial plasma afterglows. The geometrical capacitance model predicted the doublet to singlet charge ratio *R* to be 1.39^62^, determined solely by doublet geometrical capacitance. In agreement with the simulations of Sukhinin et al.^40^, the doublet charge is not described by geometrical capacitance under the collisional conditions for the charged plasma species as studied in this work.

The equivalent enclosing sphere model reported by Matthews et al.^38^ predicted a charge ratio *R* of 1.36, since the orientation-averaged equivalent radius was given by ${R}_{\sigma }=\sqrt{\left({{{\mathrm{PA}}}}/\pi \right)}\approx \ 1.36$, where ${{{\mathrm{PA}}}}=5.8{r}_{{{{{\rm{p}}}}}^{2}}$ was the average projected area of doublets^44^. Here, PA was the projected area *A*_p_ averaged over many orientations, where *A*_p_ was given by ${A}_{{{{\rm{p}}}}}={\int}_{A}\cos (\gamma )dA$, with *A* the original particle area and *γ* the angle between the plane’s normal and the line of sight to the surface. The numerical procedure to calculate PA was described by Gopalakrishnan et al.^63^. The latter model used the capacitance of the sphere with radius *R*_*σ*_ to determine the particle charge.

**Fig. 4:**
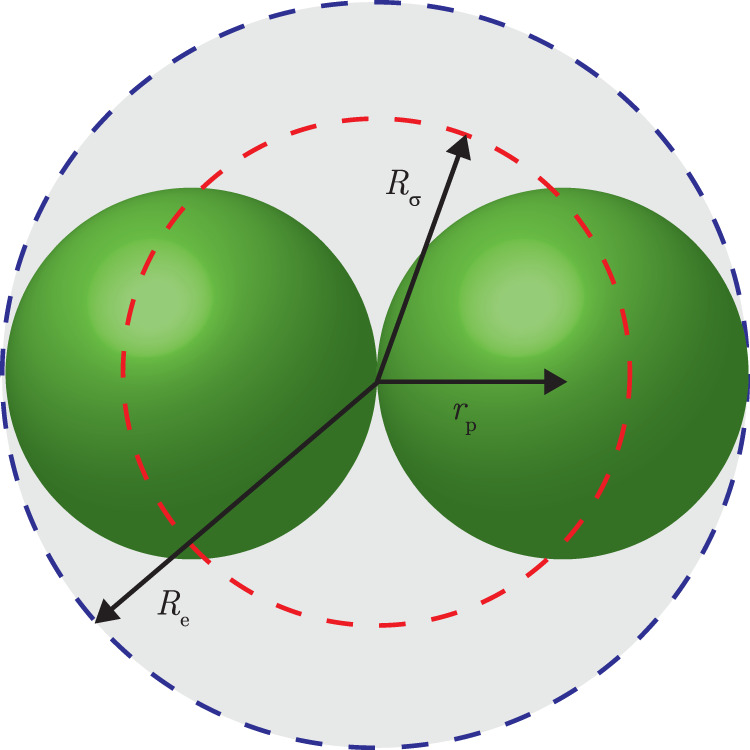
Sketch of two-particle cluster with equivalent radii. Schematic drawing of a doublet together with its smallest enclosing sphere with radius *R*_e_ = 2*r*_p_ (indicated by the dashed blue line) and the orientation-averaged equivalent sphere with radius *R*_*σ*_ = 1.36*r*_p_^38^ (indicated by the dashed red line).

On the other hand, the smallest enclosing sphere model reported by Asnaz et al. predicted *R* = 2^39^ based on the capacitance of the smallest enclosing sphere. Both the smallest enclosing and the orientation-averaged equivalent sphere are depicted in Fig. 4. It can undoubtedly be concluded that the charge of doublets in the free spatial afterglow at intermediate pressure is described by the charge on the smallest enclosing sphere.

This study measured the charge of single microparticles (singlets) and two-particle clusters (doublets) in the free spatial afterglow. The measured ratio of doublet to singlet charge was 2.0 ± 0.2. We conclude that the doublet charge was correctly described by capacitance of the smallest enclosing sphere, whereas the prediction based on the doublet geometrical capacitance proved incorrect. The manner in which these results apply to the overall theoretical framework of nonspherical particle charging in general depends on the compactness of the particle geometries^17,38^ and the flow regime of the charging species^40^. Our results are of fundamental importance in complex plasmas^3–11,38,40^, aerosol science,^21–26^ and astrophysics^14–19^, as well as in numerous applications.

## Methods

This section introduces the experimental setup, followed by the theoretical framework that is applied to detect in situ the size, mass, and cluster configuration of the particles.

### Experimental setup

**Fig. 5:**
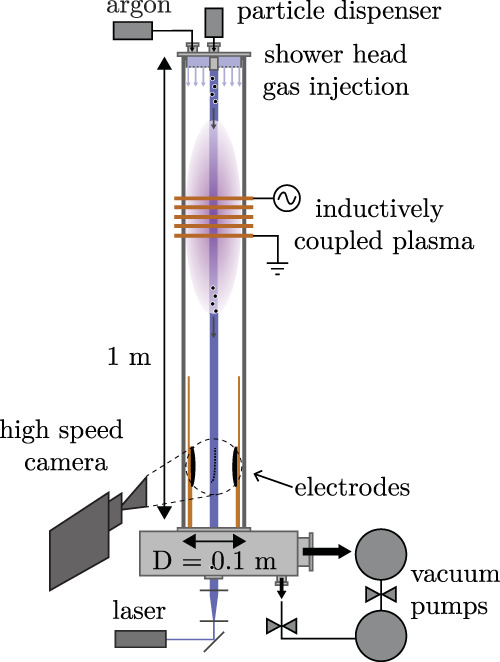
Schematic drawing of the experimental setup. Schematic drawing of the PPCI setup. Clustered microparticles traveled through a spatially limited region of plasma. The particle charge was measured at the bottom of the tube by accelerating the particles in an externally applied electric field. Figure adapted from 10.1088/1361-6595/ab8e4f, IOP Publishing. Reproduced with permission. All rights reserved.

**Fig. 6:**
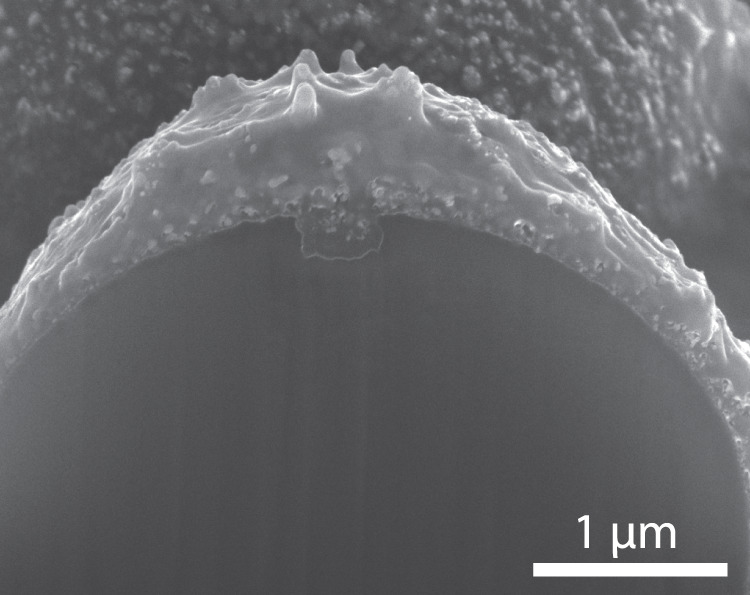
SEM image of microparticle cross-section. SEM image of microparticle cross-section of a single silver coated melamine formaldehyde microparticle including a scale bar. The cross-section is obtained by cutting the microparticle with a gallium focused ion beam (FIB). Due to the rounded beam profile of the FIB, the top part of the particle is rounded to a larger extent than the sides of the particle, which causes the coating layer to appear thicker at the top.

The experiments were carried out in a vacuum system (the plasma particle charging investigation (PPCI) setup), which is shown schematically in Fig. 5. A determining part of this system was a 1 m long square glass tube. The operating pressure was 90 Pa argon, with a background pressure of 10^−9^ Pa. At the top of the tube, particles were injected by a particle dispenser similar to the ones used in previous research^64,65^. In the dispenser’s container, the particles repeatedly collided with each other and with the container’s walls, thereby triboelectrically charging the particles^13^ and aiding the natural conformation of clustered microparticles. Figure 6 shows a cross-section of a single microparticle used in the current research. Its core consisted of MF and the outer layer of a porous silver coating with a thickness of ~100 nm. The microparticles had a diameter of *d*_p_ = (5.0 ± 0.2) μm as stated by the manufacturer, microParticles GmbH, with indicated the spread in particle diameter.

An inductively coupled plasma was generated at a frequency of 13.56 MHz using a square coil wound around the tube with a plasma power of ~3 W. The discharge was operated in the E-mode. The latter statement was based on the low plasma power applied and the light emission observed^66^. Two parallel and vertically aligned Rogowski electrodes were placed at the bottom of the setup^67,68^. A voltage difference between these electrodes created an electric field that accelerated the charged particles in a horizontal direction, from which their charge was deducted. A typical plot of the particle trajectories can be found in our previous works^36,69^. The breakdown field between the electrodes, with the plasma discharge switched on, was experimentally measured to be *E*_bd_ = 8 kV m^−1^. The field strength applied in this study (1 kV m^−1^) was much smaller than *E*_bd_. Possible reionization, therefore, had a negligible effect on both particle charge and plasma shielding. Moreover, the afterglow density did not influence the electric field distribution, as shown in our previous work^69^.

At the position of particle charge measurement, a lower limit for the plasma density was *n*_i_ = 10^11^ m^−3^^36^. Furthermore, the electrons were expected to be close to room temperature based on a simple analytical timescale, as explained in our previous publication^69^. For clarity, it should be mentioned that there are two types of plasma afterglows: temporal and spatial. The former is created by switching off the plasma discharge, while the latter is the region just outside of the active plasma region, in which the particle charging was studied in this research.

The particles were illuminated by a vertical 3-mm-thick laser sheet (central wavelength 447 nm) and their scattered light was imaged by a high-speed camera (as depicted in Fig. 5). The camera’s field of view (FoV) was ~40 mm in a horizontal direction and 32 mm vertically. Typically, the particle density was 10^6^ m^−3^, such that mutual particle interactions did not occur^36^. To obtain the respective particle charge and cluster size, the position, velocity, and acceleration of the particles were ascertained using in-house developed particle tracking software.

In contrast to our previous work^36^, there were four differences in the setup and the analysis. First, to enable cluster detection, no flow was injected at the top of the tube, as explained in the next section. To achieve vacuum purity, the plasma discharge was switched off after each measurement and pumped down to base pressure. An experiment took on average 2 min to complete.

Second, to detect both single and clustered microparticles, the time delay between the moment of particle injection and the start of camera recording varied between 10 and 21 s.

Third, the analysis required the particle trajectories to initially be detected at the top of the FoV, in addition to having a minimum length of 50 data points. This ensured that each particle was only included in the analysis once and that each trajectory contained sufficient data points for accurate fitting.

Fourth, to obtain the particles’ charge (*Q* = *m*_p_*a*_x_/*E*) from their measured horizontal acceleration *a*_x_ in the region of the applied electric field, the magnitude of this electric field *E* was calculated for each particle at the position where it had zero horizontal velocity, which was retrieved from a polynomial fit through the full imaged trajectory. To this end, a 2D electrostatic simulation was used to evaluate the electric field between the electrodes (see Fig. 2 in ref. ^36^). This simulation was performed in a geometry void of any physical obstruction above the electrodes. Using the simulation, small variations in the electric field were taken into account when obtaining the particle charge. Less than 3% of the detected particles had a nonzero horizontal velocity at each point between the electrodes and was therefore omitted from the analysis. The position at which the horizontal velocity was zero was unique for each particle, with no clear dependence on particle size. 

### Cluster detection

The technique applied in this paper to determine in situ the size, mass, and cluster configuration of the particles was based on several relations which are discussed and experimentally verified below.

The drag force on a single microparticle is described by the Epstein formula when the mean free path of the atom–atom collisions *λ*_n_ is much larger than the radius of the particle *r*_p_ (i.e., the free molecular flow regime), and when the particle speed *v*_s_ is small compared with the thermal velocity of the gas particles *v*_th,n_^70^

3 $${F}_{{{{\rm{d}}}},{{{\rm{s}}}}}=\frac{4\pi }{3}\delta {r}_{{{{\rm{p}}}}}^{2}{\rho }_{{{{\rm{n}}}}}{v}_{{{{\rm{th}}}},{{{\rm{n}}}}}{v}_{{{{\rm{s}}}}}.$$

The coefficient *δ* accounts for the microscopic mechanism of the collisions between the particles and the gas atoms. For perfect specular reflections *δ* = 1, whereas for diffuse reflections *δ* = 1.442 for a thermal nonconducting particle^70^.

The drag force on a cluster consisting of *N*_p_ particles in the free molecular regime can be quantified using the dynamic shape factor *χ*_*ν*_ as

4 $${F}_{{{{\rm{d}}}},{{{\rm{c}}}}}={\chi }_{\nu }{F}_{{{{\rm{d}}}},{{{\rm{s}}}}}({d}_{{{{\rm{ve}}}}})$$

where *d*_ve_ is the volume equivalent diameter defined as ${d}_{{{{\rm{ve}}}}}=2{N}_{{{{\rm{p}}}}}^{1/3}{r}_{{{{\rm{p}}}}}$. In the literature, numerous experiments reported on the dynamical shape factor in the continuum regime *χ*_c_^46–50^. In order to calculate *χ*_*ν*_ from *χ*_c_, the adjusted diameter was used, defined as ${d}_{{{{\rm{a}}}}}=\left({\chi }_{\nu }/{\chi }_{{{{\rm{c}}}}}\right){d}_{{{{\rm{ve}}}}}$^71^. Using the definition *ϕ* = *d*_a_/(2*r*_p_), *χ*_*ν*_ can be expressed by

5 $${\chi }_{\nu }=\frac{\phi {\chi }_{{{{\rm{c}}}}}}{{N}_{{{{\rm{p}}}}}^{1/3}}.$$

The drag force acting on clusters depends on their orientation with respect to the flow. To analyze the clusters’ orientation, the torque acting on a cylinder in the free molecular flow regime is given^72^

6 $$\tau =\frac{\pi }{4}{a}^{2}l{\rho }_{{{{\rm{n}}}}}{v}_{{{{\rm{c}}}}}^{2}{\sigma }_{{{{\rm{t}}}}}\sin \left(2\beta \right).$$

Here, *a* and *l* are the radius and length of the cylinder, *v*_c_ the velocity relative to the flow, *β* the angle between the long axis of the cylinder and the flow direction, and *σ*_t_ the tangential momentum accommodation factor.

**Fig. 7:**
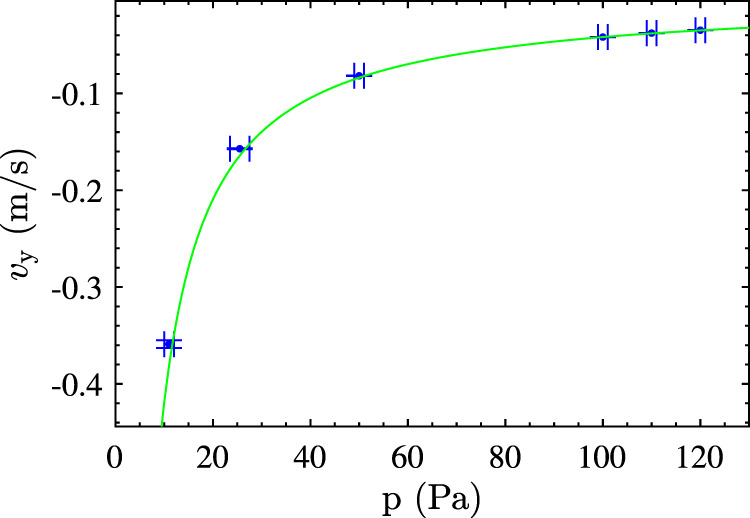
Vertical velocity of single microparticles as function of pressure. Measured vertical velocity of single microparticles as a function of the pressure together with the expected vertical velocity based on Eq. (1) in the free molecular flow regime for *δ* = 1.442 and *ρ*_*p*_ = 1.61 × 10^3^ kg m^−3^ (green line). The error bar in the pressure is determined by experimental limitations, while the error bar in the vertical velocity is represented by the standard deviation of the mean of the singlet velocity distribution.

The relations above are valid in the free molecular flow regime, which is the case for the current experiments. Figure 7 presents the measured vertical velocity of singlets as a function of the gas pressure in the absence of plasma. The other experimental conditions are identical to the ones described at the beginning of the “Methods” section. The error bars in *v*_y_ are determined by calculating the standard deviation of the mean, and in *p* by experimental limitations. Furthermore, Fig. 7 shows that the relation that could be obtained for *v*_s_ using Eq. (1) perfectly matched the experiments using *δ* = 1.442 and *ρ*_*p*_ = 1.61 × 10^3^ kg m^−3^. Therefore, it can be concluded that the drag force was indeed described by the free molecular flow relation given in Eq. (3).

## Data Availability

The data that support the findings of this study are available from the corresponding authors on reasonable request.

## Code availability

The authors used in-house developed software for particle tracking, which is created with Matlab. This code is available from the corresponding authors on reasonable request.
